# Health economic evaluation of preventive digital public health interventions using decision-analytic modelling: a systematized review

**DOI:** 10.1186/s12913-023-09280-3

**Published:** 2023-03-17

**Authors:** Oliver Lange

**Affiliations:** 1 Department of Health Care Management, Institute of Public Health and Nursing Research, University of Bremen, Bremen, Germany; 2 Leibniz ScienceCampus Digital Public Health Bremen, Bremen, Germany

**Keywords:** Cost-effectiveness, Cost-utility, Economic evaluation, Prevention, Digital public health, Digital health, mHealth, eHealth, I12, I18, C52

## Abstract

**Background:**

Digital public health (DiPH) provides novel approaches for prevention, potentially leading to long-term health benefits in resource-limited health systems. However, cost-effectiveness of DiPH interventions is unclear. This systematized review investigates the use of decision-analytic modelling in health economic evaluations of DiPH primary prevention and health promotion interventions, focusing on intervention’s design, methods used, results, and reporting quality.

**Methods:**

PubMed, CINAHL, and Web of Science were searched for studies of decision-analytic economic evaluations of digital interventions in primary prevention or health promotion, published up to June 2022. Intervention characteristics and selected items were extracted based on the Consolidated Health Economic Evaluation Reporting Standards (CHEERS). Incremental cost-effectiveness ratios (ICERs) were then extracted and price-adjusted to compare the economic evaluation results. Finally, the included studies’ reporting quality was assessed by building a score using CHEERS.

**Results:**

The database search (including search update) produced 2,273 hits. After removing duplicates, 1,434 titles and abstracts were screened. Of the 89 studies meeting the full-text search criteria, 14 were ultimately reviewed. The most common targets were physical activity (five studies) and weight loss (four). Digital applications include text messages, web-based inventions, app-based interventions, e-learning devices, and the promotion of smartphone apps. The mean ICER of the 12 studies using quality-adjusted life years (QALYs) is €20,955 per QALY (min. − €3,949; max. €114,211). The mean of reported CHEERS items per study is 81% (min. 59%; max. 91%).

**Conclusions:**

This review only includes primary prevention and health promotion, and thus excludes other DiPH fields (e.g. secondary prevention). It also focuses on decision-analytic models, excluding study-based economic evaluations. Standard methods of economic evaluation could be adapted more to the specifics of DiPH by measuring the effectiveness of more current technologies through alternative methods, incorporating a societal perspective, and more clearly defining comparators. Nevertheless, the review demonstrates using common thresholds that the new field of DiPH shows potential for cost-effective preventive interventions.

**Supplementary Information:**

The online version contains supplementary material available at 10.1186/s12913-023-09280-3.

## Introduction

Digital public health (DiPH), i.e. the use of digital means to address Public Health functions like health promotion or its governance [1, 2], is a new, expanding field. The potential benefits and advantages of DiPH could support the transition from cure to prevention, the empowerment of people and patients, and progress towards safer, cheaper, and more efficient health care management delivery [3].

Established concepts of digitalization in health care and public health, such as eHealth, mHealth, and digital health, target the individual level. By contrast, DiPH targets the population level [2]. While DiPH can be defined broadly to include health protection, health promotion, primary, secondary and tertiary prevention as well as cure [1], it can also be associated with a focus on disease prevention, health promotion [4], following the wide-spread view that ‘prevention is better than cure’ [5].

In this review, the term ‘digital’ is used in its broadest sense to refer to the use of information and communications technology. Examples of digital interventions are health apps, SMS reminders, web-based applications, and electronic devices. Based on the NICE Evidence Standards Framework for Digital Health Technologies [6], this study investigates interventions targeting preventive behaviour change, including changes in user behaviour related to health (e.g., smoking, alcohol consumption). It excludes interventions used to treat diagnosed conditions.

While the number of digital applications in (public) health is rising, it remains unclear whether they meet the ambition of providing cost-effective or even cost-saving care. Given that public health budgets are limited, coverage decision-makers need to assess not only effectiveness, but also the cost-effectiveness of new DiPH interventions to appraise whether they should be included into the reimbursement schemes. A formal method to do so is economic evaluation, which compares two or more interventions in terms of costs and consequences [7].

There are different types of economic evaluations. In this review, cost-effectiveness analysis (CEA) is understood to be a comparison of the relationship between costs and single or multiple health effects that are common to two or more alternatives [8]. Cost-utility analysis (CUA) includes the concept of utilities, using generic outcomes like disability-adjusted life years (DALYs) or quality-adjusted life years (QALYs). Other types include cost–benefit analysis, which also expresses costs and health effects in monetary units, or cost-minimisation analysis, which compares costs while health effects are assumed to be equal.

CEA and CUA can be study- or model-based: study-based economic evaluations generally elicit data through a relatively short-term concrete trial, whereas decision-analytic models combine data from different sources. There are advantages to economic evaluations using decision-analytic modelling: they allow synthesizing various input data, including different comparators, extrapolating costs and effects over time, and systematically accounting for the uncertainty of available evidence for the specific decision problem [9]. Therefore, model-based economic evaluations can forecast costs and health outcomes over a long time. Generating evidence about the costs and effects of preventive interventions is generally challenging, especially given the long time horizon over which effects manifest. This challenge is even greater for digital interventions, characterized by high innovation dynamics. Therefore, decision-analytic modelling may be particularly suited for assessing new DiPH interventions.

Economic evaluation allows decision-makers to compare new DiPH interventions with alternative uses of the limited resources. In cost-effectiveness analyses, one standard to do so is to calculate the incremental cost-effectiveness ratio (ICER) which can be used to measure, for example, the cost per kg weight loss. Analogously, in cost-utility analyses, it is the calculation of an incremental cost-utility ratio (ICUR), which based on the difference of costs divided by the difference of QALYs and can be exemplarily expressed as cost per QALY gained ([10], p. 41). Following the established definition [8], in the following sections this will be summarised under the term ICER. The ICER can then be compared to a threshold of cost-effectiveness, corresponding with the social willingness to pay for a QALY or the opportunity costs in terms of the cost-effectiveness of interventions that are replaced by the new DiPH interventions. While this allows for a theoretically sound economic assessment, it has to be noted that the choice of threshold value is a contested topic and there are varying thresholds in the literature (e.g. [11, 12]).

Widely used guidance for reporting economic evaluation are provided by the Consolidated Health Economic Evaluation Reporting Standards (CHEERS) [13] which recommend including specific items into the title and abstract, introduction, methods (e.g. comparator, time horizon, choice of model), results (e.g. study parameters, characterizing uncertainty), and discussion. The CHEERS were originally co-published by 10 academic journals that frequently publish health economic evaluations [13, 14] and these standards are acknowledged as key reporting guidelines by the EQUATOR Network. Therefore, they are used in this review as methodological standard for reporting health economic evaluations.

To make evidence-based decisions in DiPH, decision-makers and researchers need an overview of the existing health economic evidence. Specific fields of digital health have already been studied. Investigated topics include preventive interventions and treatment of conditions and diseases such as diabetes [15] and depression [16]. The economic evaluation of digital interventions for primary prevention and health promotion has already been investigated for older people but not the broader population [17]. While the cost-effectiveness of internet-based interventions up to 2008 has been reviewed [18], the current review is updated and systematized. Digital prevention and health-promotion interventions have recently been investigated but without focus on cost-effectiveness [19].

Therefore, the aim of this systematized review is to identify and investigate the use of health economic evaluation using decision-analytic modelling to evaluate DiPH primary-prevention and health-promotion interventions, focusing on (1) which DiPH interventions have been evaluated by now, (2) the evaluation methods, (3) the results on the cost-effectiveness of DiPH, and (4) the studies’ reporting quality.

## Methods

The systematized review is reported in accordance with the PRISMA 2020 guidelines [20]. To find a homogenous and comparable group of studies, four main eligibility criteria were set: (i) model-based (not study-based) economic evaluation, in the expectation of long-term analysis and to enable investigation of unique study types; (ii) primary-prevention or health-promotion intervention, supporting the transition from cure to prevention; (iii) use of information and communications technology in the intervention excluding, simple phone calls; and (iv) original study.

PubMed, CINAHL, and Web of Science were searched for potentially relevant studies on 4 December 2020 and updated in June 2022. The search strategy linked the concepts of economic evaluation and digital (health) technologies. Based on the requirement of the CHEERS [13] item ‘Title’, that the title had to include a term of economic evaluation, titles must include one or more of various terms referring to economic evaluation (e.g. cost-effectiveness). Using the Boolean operator ‘AND’, the search included variations of ‘digital health’ and different digital technologies/applications (e.g. web-based) in the title or abstract (see Additional file 1). In addition, reference tracking was performed for known literature reviews.

The first step in the selection process used Microsoft Excel to eliminate duplicates followed by the title abstract screening. As the abstract may not reveal whether a study uses a model or a different method of economic evaluation title–abstract screening was based on broader criteria than in the full-text investigation. Specifically, studies were selected for full-text investigation if they:

a reported the quantitative results of a health economic evaluation or compared the costs of two alternative interventions;
b focused on a DiPH intervention involving information and communications technology; and
c evaluated an intervention targeting primary prevention or health promotion.

Studies whose abstract was unavailable and whose title clearly did not comply with the criteria were excluded. Full texts were excluded if the study did not meet all of eligibility criteria (i)–(iv). Titles and abstracts were double-screened independently by OL and LB or by OL and WT. The two reviewers sought consensus in cases of disagreement; where agreement could not be reached, a senior researcher (WR) decided on inclusion. Two reviewers (OL and LB) independently investigated full texts and checked their eligibility. Disagreements were resolved through an iterative process by one reviewer (OL), who conducted extraction and quality reporting, supported by advice from WR on ambiguous cases.

With the search update OL performed title–abstract screening and full-text investigation and extraction following the same process and criteria of the original search.

The first step of assessing the identified studies focused on the interventions they evaluated. The following were extracted: the aim of the intervention (e.g. increasing physical activity); the primary digital component (e.g. smartphone application); any complementary web tools (to establish the complexity of the intervention); any requirement for face-to-face meetings; and more generic items like target population and location.

The second step assessed the studies’ evaluation methods. Selected items from the CHEERS and additional items of interest were operationalized and extracted to describe the characteristics and commonalities of included studies: type of economic evaluation, type of model, health outcome, time horizon, discount rate for outcome and costs, and measurement of effectiveness. Reported limitations of the studies and other relevant information in the discussion sections were also extracted and named as "self-reported limitations" in the results section of this review.

The third step assessed the studies’ results. Specifically, for every study comparing a digital intervention and a base-case scenario, the ICER was extracted. This allowed comparing the results of economic evaluations. Only ICERs resulting from the main investigation were considered, thus excluding ICERs for other scenarios (e.g. uncertainty analysis results). OECD purchasing power parities (PPP) were used to adjust ICERs to a common euro price [21]: after first converting each ICER by the PPP value of the corresponding year into US dollars, the US dollar prices were then converted to euros through multiplying by the ‘Euro area (19 countries)’ PPP value [21]. For comparability, only ICERs with QALY health outcomes were included.

Finally, to assess the studies’ reporting quality, every CHEERS item [22] was assessed for each economic evaluation, producing a total quality score. Items labelled ‘If applicable’ were not evaluated, thus ensuring that scores were always calculated in the same way. If an items required an additional reason, this had to be supplied for the item to be considered fully reported. If an CHEERS item was reported almost completely, it was assigned a score of 1; CHEERS items fulfilled partially or not scores of 0.5 or 0, respectively. The appendix was recognized as part of the study, but information from cited references was not included. For each study, the overall score was calculated as the sum of item values divided by the total number of CHEERS items.

## Results 

**Fig. 1:**
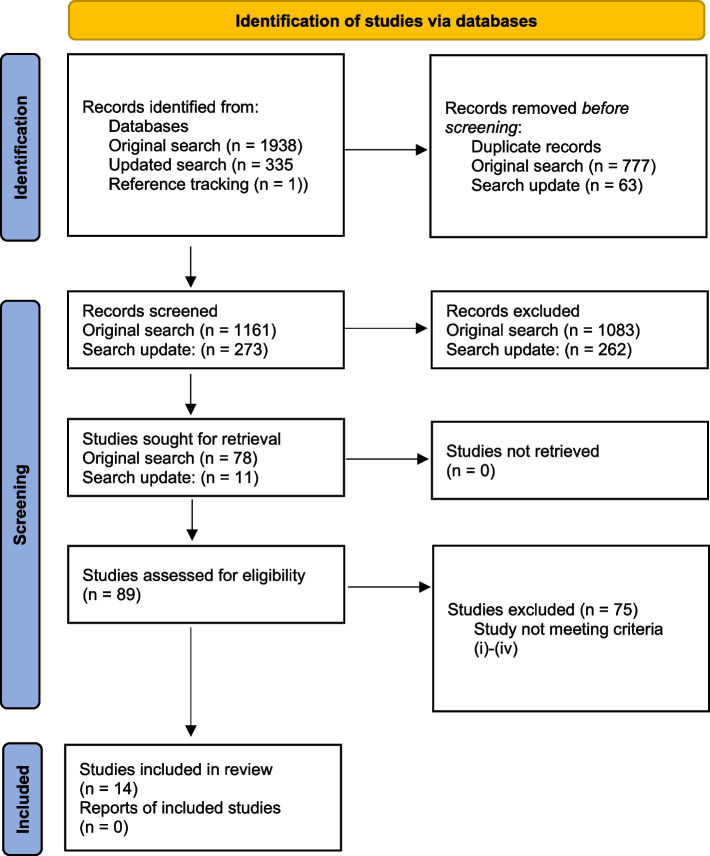
Modified flow diagram based on PRISMA 2020 [20]

The database search found 1,938 studies. After removing 774 duplicates, 1,161 titles and abstracts were screened. The 78 studies that fulfilled the criteria were investigated in a full-text search. In the updated search, 335 titles and abstracts articles were screened, and 11 full texts were investigated. Finally, data were extracted from the 14 studies that met the eligibility criteria. In Fig. 1, the modified PRISMA flow diagram [20] shows the review process.

### Studies that might appear to meet the inclusion criteria

PRIMSA 2020 [20] requires that details are provided for studies that might appear to meet the eligibility criteria but are excluded. This was the case for seven studies, covering:

1. the application of a digital decision tool for economic evaluation of a non-digital intervention [23];
2. a cost-minimization analysis that did not demonstrate at least equal health outcomes [24];
3. an intervention using telephone counselling, which is not considered digital [25];
4. an intervention to prevent suicide but not suicidal thoughts, which did not meet this review’s definition of prevention [26];
5. an alcohol intervention with mainly therapeutic and secondary prevention elements, rather than primary prevention [27];
6. a diabetes-prevention intervention targeting individuals with a weight-related risk factor (i.e. hypertension) – this constitutes secondary prevention as screening is required to detect the risk factor [28]; and
7. a population of individuals with high risk of cardiovascular disease, based on either a history of the disease or a risk equation, and thus involving screening [29].

### Assessed DiPH interventions

**Fig. 2:**
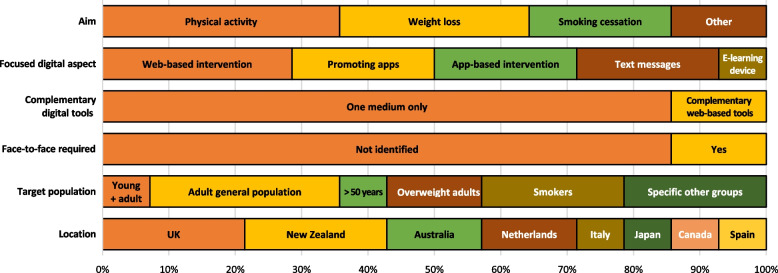
Assessed digital public health interventions

Figure 2 shows the characteristics of assessed DiPH interventions (for details of individual studies, see Additional file 2). The interventions subject to economic evaluation pursued different goals: increase physical activity [30–34], lose weight [35–38], stop smoking [39–41], change health-related behaviours [42], and manage menstrual health [43]. Thus, most interventions aimed to prevent typical chronic diseases such as diabetes or heart disease.

The digital tools for pursuing these goals were also heterogeneous. Of the five physical-activity interventions, the first was particularly complex, combining text messages with complementary online exercises, a Facebook group, and face-to-face meetings to increase the physical activity of women with young children in Australia [30]. The second was web-based/internet intervention and targeted the Australian population aged over 15 [31]. The third physical-activity intervention delivered the same advice via a website and by mail, while also providing exercise videos; it targeted people over 50 [32]. The fourth intervention was a commercial smartphone app [33], while the fifth promoted existing apps to increase physical activity, targeting adults in New Zealand [34].

Of the four weight-loss interventions, the first promoted existing apps and targeted overweight or obese adults in New Zealand [37]. The second intervention was a mass-media campaign to promote existing smartphone apps for weight loss in New [38]. The third weight-loss intervention was purely app-based and targeted 15–64 year-olds in Italy [35]. The fourth employed e-learning devices and was assumed by the study to have a target population aged exactly 50 with BMI > 30 [36].

There were also three smoking-cessation interventions: two were based on text messages and respectively targeted smokers in the United Kingdom [39] and Spain [40], while the third was a web-based intervention with personalized feedback targeting smokers in the Netherlands [41]. Another intervention involved an online portal aiming to induce general behavioural change among young people starting their university studies [42]. Finally, one intervention entailed smartphone app-based menstrual management, aimed at preventing depression and dysmenorrhea. The economic evaluation of DiPH is thus no homogeneous field of investigation on which general conclusions can be drawn easily but incorporates a rich variety of very different interventions.

### Evaluation methods

**Fig. 3:**
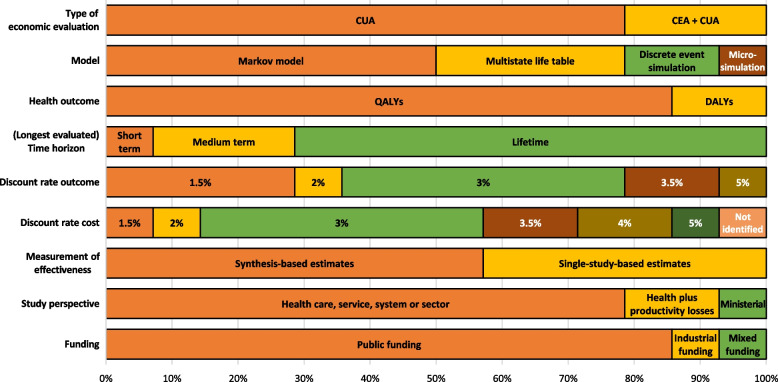
Evaluation methods

Figure 3 shows the methods of economic evaluation. Studies typically compared the digital intervention with doing nothing or ‘business as usual’, and it was sometimes unclear what level of digital intervention the ‘usual’ scenario entailed or how widely used digital applications already are in the analysed healthcare system.

Most studies were conducted from a health care perspective [30–32, 34–39, 41], expressly identified as a health service, sector, or system perspective. Two studies additionally included societal costs (productivity losses [40, 43]), and one gave a ministerial perspective [42]. As some of the studies acknowledged, focusing on the health care perspective overlooks other costs such as productivity losses [34, 35] or greenhouse gas emissions [37].

The decision-analytic model in all the studies included CUA with QALYs (12 studies) or disability-adjusted life years (DALYs; two studies) as the health outcome. Three studies also conducted CEA, expressing the results as cost per smoking quitter [39], life years gained [42], or disease incidence [32]. Seven studies used Markov models; four employed multistate life table models ([34, 37, 38] used the same model); two used discrete event simulations; and one employed the OECD Strategic Public Health Planning for NCDs model, which forecasts the costs and health outcomes of different hypothetical public health measures up to 2050 [35].

Time horizon, which was expected to be long-term for decision-analytic studies on (chronic) disease prevention, was relatively short for two studies – two years [30] and five years [33] – but longer for all the others (e.g. lifetime perspective). Two studies used specific time horizons: one for women up to 45 years, the other for the years from 2019 to 2050. Although these analytic time horizons are long, the effects of the interventions were partially assumed to be short or are based on evidence of short-duration trials only. For example Mizdrak et al. [34] reported in their limitations that there was no evidence that the impact of the intervention persists for more than one year, and thus studies on long-term effects are needed.

Model-based economic evaluations include estimates of the effectiveness of interventions based on one (single study) or more studies (synthesis based). In this review, eight of the included models based their effectiveness estimates on a synthesis of studies (e.g. meta-analysis), whereas the other six based estimates on a single study. Several limitations were self-reported about the effectiveness studies used: For example, Cleghorn et al. [37] mentioned that effectiveness could be underestimated if data on smartphone app download rates are lacking, or if spillover effects within households and technological improvements over time are disregarded [37]. Further, evaluations of similar interventions (e.g. weight-loss apps) may find different levels of effectiveness [34, 37].

Various assumptions were made to forecast long-term effects with decision-analytic models. Self-reported limitations of these assumptions mainly concerned the under- or overestimation of effects due to the modelling. Therefore, effects could be underestimated if the impact of physical-activity interventions on weight loss is not considered or only certain diseases are modelled (e.g. disregarding mental health [34]). Moreover, if included diseases are assumed to be independent [34], the model does not reflect that an individual with one disease may have a greater risk of contracting another (e.g. cardiovascular disease increases the risk of diabetes). One study reported that dynamics in diseases (e.g. different health states within an activity level) were not considered – only inactive and active states [30]. Thus, with a small increase in physical activity, no change could occur. Further reported assumption limitations include failing to consider that the risk of contracting a disease could be irreversible or only decline a long time after behavioural change [31], or modelling future risk independently of past behaviour [32]. In another study modelling the promotion of existing apps, it was assumed that a temporary mass-media campaign for one year would stop, thus neglecting potential effects if it continued [37]. Even if decision-analytic modelling may appear as a well-suited approach to cope with the difficulties in the economic evaluation of DiPH, the existing models also reveal various methodological challenges.

### Results of health economic evaluations

Table 1 shows the included studies’ results, ordered by ICER. They are given in different currencies and price years. The calculated mean is €20,955 per QALY (studies using DALYs are not included). However, this value should be handled with caution and has low significance for decision-making, since it is based on a small number of heterogenous economic evaluations. The lowest ICER was found for a smoking-cessation study [41] resulting in cost-savings (− €3,949 per QALY). By contrast, the highest ICER was calculated for a weight-loss intervention [36] delivered by an e-learning device (€114,211 per QALY). Regarding intervention aim, those targeting smoking cessation had a lower ICER on average relative to physical-activity interventions. Jones et al. [38], Cleghorn et al. [37] and Mizdrak et al. [34] all evaluated interventions promoting existing apps, but the first study reported a much lower ICER than the other two. This difference may be explained by the use of different effectiveness studies and amounts of available evidence.

**Table 1:** Study results expressed in ICER

Authors	Intervention	Delivery	ICER	Currency per Health-outcome	Country	Price-year	ICER € per QALY	Note
Cheung et al	Smoking cessation	Web-based	-4,306.50^a^	Euro per QALY	Netherlands	2016	-3,949.43	Price-year not identified Literature search has ended in 2016 Only lifetime horizon considered; ^a^ICER = 602.91 € / 0.14 QALY
Jones et al	Weight loss	Promoting apps -	-3348.07^b^	NZ$ per QALY	New Zealand	2011	-1,757.40	Price-year referred to Cleghorn et al.; ^b^ICER = -606,000 / 181 QALY
Guerriero et al	Smoking cessation	Text messages	-1431,3448^c^	GBP per QALY	United Kingdom	2009–2010 (2010 used)	-1,616,89	Only weighed average ^c^ICER = -41,509 / 29 QALY
Cobos-Campos et al	Smoking cessation	Text messages	1327	Euro per QALY	Spain	2018	1,496.81	Only Woman & Health system perspective considered
Burn et al	Physical activity	Text messages	8,608	AUS$ per QALY	Australia	2014	4,458.14	
Rondina et al	Physical activity	App-based	11,113	CAD per QALY	Canada	2018	6,525.23	
Peels et al	Physical activity	Web-based	10,100	Euro per QALY	Netherlands	2011	9,423.44	Only lifetime scenario
Song et al	Menstrual management	App-based	-1,914,285^d^	Yen per QALY	Japan	2017	13,132.01	^d^ICER = -134,000 / 0.07 QALY
Kruger et al	Behaviour change (mixed)	Web-based	22,844	GBP per QALY	United Kingdom	2012	25,186.97	Only the "Implementation at University of Sheffield"-scenario
Cleghorn et al	Weight loss	Promoting apps	79,700	NZ$ per QALY	New Zealand	2011	41,834.45	
Mizdrak et al	Physical activity	Promoting apps	81,000	NZ$ per QALY	New Zealand	2011	42,516.82	
Miners et al	Weight loss	E-learning device	102,000	GBP per QALY	United Kingdom	2009	114,211.27	Only Scenario A

^a-d^ICER Calculated by article author on the basis of existing information in extracted study. All other ICERs were extracted directly from the study

### Reporting quality of health economic evaluations

**Fig. 4:**
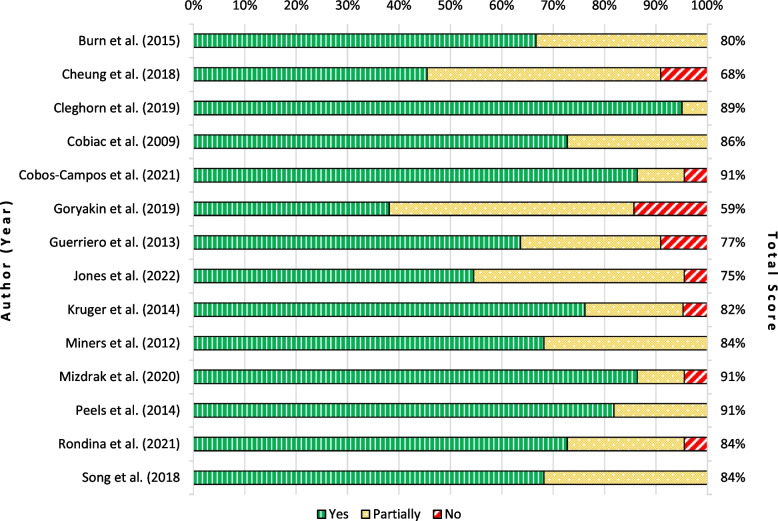
Reporting quality per study (in terms of item reported – yes, partly, or not)

Figure 4 shows the assessment of reported items on the CHEERS checklist. The mean of reported CHEERS items per study is 81%, with a minimum of 59% and maximum of 91%. One reason for the high number of partially reported items is studies reporting, for example, the discount rate or time horizon but not stating why this is appropriate. Some studies do not provide all the required information in the abstract, or do not refer to common standards. Only a few items are unreported or only insufficiently reported.

**Fig. 5:**
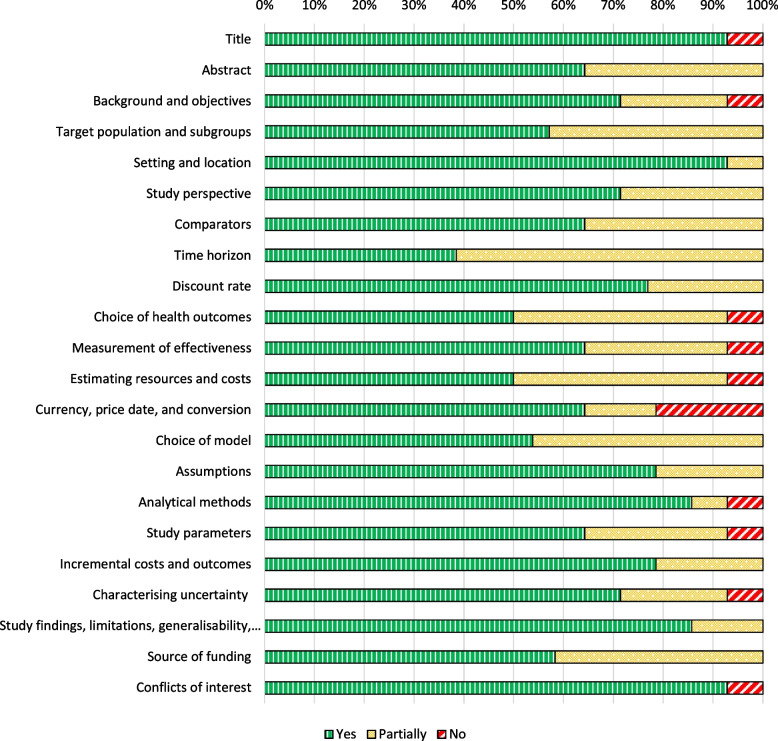
Reporting quality per CHEERS item (in terms of item reported – yes, partly, or not)

Figure 5 shows the assessed fulfilment of each CHEERS item. All studies reported a time horizon but few stated why the chosen horizon was appropriate. Moreover, for the estimation of resources and costs, sources were named without an accompanying explanation of why they were sufficient or how they were found.

## Discussion

This review gives an overview of the use of decision-analytic modelling to evaluate digital interventions in primary prevention and health promotion. Of the 12 studies reporting outcomes in terms of QALYs, nine were found to have an ICER below €50,000 per QALY, while the overall mean was €20,955 per QALY. However, no inferences should be drawn on the cost-effectiveness of DiPH interventions: (1) the mean is based on a small sample of heterogeneous studies; and (2) the frequently cited threshold value of €50,000 per QALY is contested, and cost-effectiveness should always be assessed by the relevant health care decision-makers, comparing against other adopted or rejected interventions [11, 44]. Moreover, the results are far from conclusive, and it is not possible to determine which digital technology is the most cost-effective.

### General interpretation of results considering other evidence

Other reviews of economic evaluations with a similar research question do not provide detailed information on the cost-effectiveness of DiPH preventive interventions. In 2009, Tate et al. [45] reviewed the cost-effectiveness of internet-based interventions. They identified one health-promotion and one obesity-management intervention that could be classified as primary prevention, but these two studies did not use decision-analytic models. It can also be assumed that technological progress since 2009 has led to new digital interventions. In 2015 [46], a systematic review of economic evaluations focused on telemedicine, eHealth, and mHealth, considering public health fields such as secondary prevention (in the form of screening). However, no methods of evaluating primary-prevention or health-promotion interventions are included or analysed. In 2020, Ghani et al. [47] conducted an overview of the cost-effectiveness of mHealth for older adults, but did not include any primary-prevention interventions (target groups were patients or persons with diseases). One systematic review [48] investigated economic evaluations of eHealth for older adults. They identified Peels et al. [32] as the only study making a primary-prevention intervention – all other studies involved interventions against existing diseases.

In 2017, Iribarren et al. [49] reviewed economic evaluations of mHealth interventions in general: only a few identified studies involved typical primary-prevention or health-promotion interventions (targeting physical activity, vaccination, and obesity). Only two economic evaluations included in this review, [30] and [39], also feature in Iribarren et al.’s synthesis of results. This could be explained by the latter review being conducted five years earlier and including only mHealth interventions, as well as it was not restricted to studies using decision-analytic modelling. While Law et al. [50] conducted a review of telehealth-delivered diet and exercise interventions, it included only studies of individuals with at least one health condition.

In the absence of a prior review of cost-effectiveness focused on DiPH preventive interventions, meaningful comparison of potential cost-effectiveness is only possible with individual economic evaluation studies.

### Limitations of the included evidence

Depending on the model used, assumptions made, and data collected, the included studies have several limitations, which are frequently reported in the studies. This section discusses the particularities of economically evaluating DiPH interventions, considering the use of effectiveness studies, selection of perspective, and choice of comparators. Limitations reported by the studies themselves were reported in the results section.

Economic modelling is based on effectiveness studies, which must be designed, conducted, evaluated, and published. It is well known that this scientific process takes time. However, digital applications are evolving rapidly, as illustrated by the evolution of cell phones from simple devices to smartphones equipped with various sensors and the ability to connect to servers or other devices. Yet this review has shown that many DiPH interventions still rely on web-based tools or text messages. At this stage, the evaluated interventions seem to lag far behind technical developments, with no just-in-time adaptive interventions identified, nor any built around sensors. This limits the value of effects estimates based on a single study and especially those based on synthesized results: meta-analyses may include old technology and, compared to the studies they analyse, have a greater risk of presenting outdated effectiveness estimates. To avoid this, other study designs could be used (e.g. n-of-1 trials [51]) that allow collecting data on effectiveness more quickly. No study applying such a design was identified in this review.

Only two identified studies offer a societal perspective, and both are limited to productivity losses. However, in the economic evaluation of prevention interventions (this also includes DiPH), all societal costs and benefits should be taken into account [52]. Moreover, given the importance of addressing climate change in health care and public health, there may be a need to extend the perspective beyond monetary costs to all members of society to include detrimental effects to the environment which cannot easily be monetarized. No conceptual framework to obtain such a broad perspective in economic evaluations was applied in the studies nor appears to be available in the health economic literature.

Another limitation of the evidence is that most investigated economic evaluations do not explicitly identify the comparator for the DiPH intervention. Although referring to ‘usual care’ or ‘business as usual’, they do not clearly describe the digital environment in the investigated country. It may be assumed, for example, that states with higher (vs. lower) levels of digitalization have higher public openness to digital health services, more scalable costs, and more effective interventions. This review’s findings reveal a tendency to name the digital environment but not describe it in detail. The mERA checklist could provide a solution to this problem by requiring the definition of comparators with respect to the digital environment (e.g. infrastructure, technology platform, and interoperability) [53].

### Limitations of the review processes

This review has several typical limitations. It only includes research literature written in English, and only three databases were searched. When unclear information was found, authors were not contacted to request clarification [20]. Only original studies were included. The terms “reports” and “studies” are taken to be synonyms, although PRISMA requires to distinguish between reports and studies. The search strategy could have been modified to add the term ‘decision-analytic’ to the concept of economic evaluation. The search strategy restricts the terms of economic evaluation to the title because CHEERS requires that authors state the type of economic evaluation in the article title. This search strategy was chosen because (1) the broad complementary search term to identify DiPH interventions had to be limited without losing evidence and (2) a large number of complementary exploratory searches indicated that combining this strategy with additional reference tracking is likely to identify all relevant decision-analytic economic evaluations.

The criterion that studies had to involve a primary-prevention or health-promotion intervention was sometimes difficult to apply. Smoking, drinking alcohol, obesity, and physical inactivity were considered as risk factors, not emerging diseases. However, studies whose target population was a (pure) patient group were excluded. While interventions targeting individuals at higher risk, as determined by a risk score, were considered in the screening phase of this review, these studies were subsequently excluded as secondary prevention. The requirement for studies to include a full economic evaluation also led to the exclusion of single individual studies, such as those that did not apply a model but only calculated alternative scenarios based on different input parameters (e.g. [54]). The search strategy also excluded all study-based economic evaluations, thereby missing possible insights from longitudinal studies or evaluations with a lifetime horizon. However, the above-mentioned advantages and need for comparability were considered to justify restricting the review to decision-analytic economic evaluations.

The mean ICER only takes the base-case results in each study into account. However, the base-case scenario in Kruger et al. [42] showed an ICER of £22,844 per QALY for a rollout restricted to one university, while an additionally evaluated rollout to other universities resulted in an ICER of £1,545 per QALY. This indicates the possibility that an intervention yielded a much lower ICER and was not considered in the results of this review because not all possible scenarios were included in an analysis in the synthesis. It should also be noted that the ICER can be misinterpreted (for example if it is high but incremental QALYs are low, or if it is negative), hence there are reasons to consider costs and outcomes separately [55]. Although prices were adjusted to PPP, no adjustment was made, as technological progress had not been considered. However, technologies can be expected to become more efficient or more widespread. Because of potentially falling IT costs, health care inflation rates were considered inappropriate, so it was deemed best to assume stable prices.

Regarding the quality assessment based on CHEERS items, a high score does not necessarily imply a high-quality evaluation as the instrument only assesses reporting transparency. Conversely, a low score does not necessarily indicate a low-quality study. CHEERS compliance was interpreted strictly, and missing reasons for choosing items resulted in a lower score. Moreover, referring to a source without presenting the respective content was not considered sufficient, thus decreasing the score. For example, merely referencing a very detailed RCT without describing why the intervention, comparator, and target population were selected could not be considered when assessing the fulfilment of an item. However, any reference to basic guidelines as a rationale for decisions (such as the discount rate) was sufficient. While the CHEERS consolidate other established checklists, more model-specific reporting guidelines could have been used instead (e.g. [56]). On balance, though, the widely known CHEERS were considered the most suitable yardstick for assessing quality. In light of the PRISMA statement, this review did not conduct subgroup or robustness analysis or certainty assessment because the sample was too small for these complementary analyses.

Although selected self-reported limitations were included in the results section, a comprehensive critique of the models is only possible within more detailed analyses. The heterogeneous nature of interventions assessed by the evaluations in this review precludes such analysis here.

### Implications of the results for practice and policy

This review suggests that some DiPH preventive interventions are potentially cost-effective. In particular, one reviewed smoking intervention yielded cost savings, with intervention costs lower than disease costs. DiPH interventions thus provide a new and potentially promising field of prevention that might, in some cases, even incur cost savings to health care payers.

However, they warrant economic evaluation. Decision-analytic modelling may be a particularly suited methodology to do so: first, it is comparatively easy to develop a large number of different versions that are more easily assessed by models than by clinical trials; and second, the effects of preventive interventions have a potentially long time horizon. However, this review also revealed multiple methodological difficulties of identifying appropriate estimates of effectiveness for DiPH technologies with short technology life cycles. There thus remains the need to generate sound evidence of effectiveness.

### Implications of the results for future research

This systematized review illustrates that the health economic evidence underscores the potential health effects and (disease) costs of DiPH prevention interventions. However, it also demonstrates that the evidence for cost-effectiveness in this field is still weak, highlighting the need for further studies to assess the cost-effectiveness of multiple forthcoming interventions.

Recent methodological studies identify specific challenges for evaluating DiPH, such as the plurality of outcomes, including not only individual health benefits but also a broader societal perspective, for example by measuring the carbon footprint of digital health interventions [57]. Equity impacts may also be particularly important in this field. None of the included studies fully accounted for these challenges.

While this systematized review provides an initial reference point of existing economic evaluations for digital primary prevention, further research is needed on what such evaluations should include and how to address the methodological challenges that were identified.

## Conclusion

Based on common thresholds, there are DiPH interventions which are potentially cost-effective. However, the economic evidence in this field remains weak. Also, the interventions identified in this review are too heterogeneous and digital technology life cycles are too short to draw general conclusions on the cost-effectiveness of DiPH.

## Supplementary Information

**Additional file 1.**

**Additional file 2.**

## Data Availability

The search strategy is presented in Additional file 1, while study details extracted from included studies are available in Additional file 2.

## Abbreviations

CHEERS: Consolidated Health Economic Evaluation Reporting Standards
DALY: Disability-adjusted life year
DiPH: Digital public health
ICER: Incremental cost-effectiveness ratio
PPP: Purchasing power parities
QALY: Quality-adjusted life year

## References

[1] World Health Organization. Essential public health functions, health systems and health security: developing conceptual clarity and a WHO roadmap for action. 2018. https://apps.who.int/iris/handle/10665/272597. Accessed 15 Sept 2022.

[2] Wienert J, Jahnel T, Maass L (2022). What are Digital Public Health Interventions? First Steps Toward a Definition and an Intervention Classification Framework. J Med Internet Res.

[3] Odone A, Buttigieg S, Ricciardi W, Azzopardi-Muscat N, Staines A. Public health digitalization in Europe. Eur J Public Health. 2019; 10.1093/eurpub/ckz161. 10.1093/eurpub/ckz161 PMC6859512 31738441

[4] Zeeb H, Pigeot I, Schüz B, Leibniz-WissenschaftsCampus Digital Public Health B. [Digital public health-an overview]. Bundesgesundheitsblatt - Gesundheitsforschung - Gesundheitsschutz. 2020; 10.1007/s00103-019-03078-7. 10.1007/s00103-019-03078-7 31919531

[5] Department of Health and Social Care. Prevention is better than cure: our vision to help you live well for longer 2018. https://assets.publishing.service.gov.uk/government/uploads/system/uploads/attachment_data/file/753688/Prevention_is_better_than_cure_5-11.pdf. Accessed 17 Aug 2021.

[6] National Institute for Health Care Excellence. Evidence standards framework for digital health technologies. 2019. https://www.nice.org.uk/corporate/ecd7. Accessed 17 Aug 2021.

[7] Claxton K, Drummond MF, Sculpher M, Stoddart GL, Torrance GW (2015). Methods for the economic evaluation of health care programmes.

[8] Drummond MF, Sculpher MJ, Claxton K, Stoddart GL, Torrance GW. Methods for the Economic Evaluation of Health Care Programmes. Oxford: Oxford: Oxford University Press; 2015.

[9] Briggs A, Sculpher M, Claxton K (2006). Decision modelling for health economic evaluation.

[10] Edwards RT, McIntosh E. Applied health economics for public health practice and research: Oxford University Press; 2019.

[11] Grosse SD. Assessing cost-effectiveness in healthcare: history of the $50,000 per QALY threshold. Expert Rev Pharmacoecon Outcomes Res. 2008; 10.1586/14737167.8.2.165. 10.1586/14737167.8.2.165 20528406

[12] McCabe C, Claxton K, Culyer AJ. The NICE cost-effectiveness threshold: what it is and what that means. Pharmacoeconomics.2008; 10.2165/00019053-200826090-00004. 10.2165/00019053-200826090-00004 18767894

[13] Husereau D, Drummond M, Petrou S, Carswell C, Moher D, Greenberg D, et al. Consolidated Health Economic Evaluation Reporting Standards (CHEERS) statement. Value Health. 2013;10.1016/j.jval.2013.02.010. 10.1111/1471-0528.12241 23565948

[14] Husereau D, Drummond M, Augustovski F, de Bekker-Grob E, Briggs AH, Carswell C, et al. Consolidated Health Economic Evaluation Reporting Standards 2022 (CHEERS 2022) statement: updated reporting guidance for health economic evaluations. BMJ. 2022; 10.1136/bmj-2021-067975. 10.1111/1471-0528.17012 35014160

[15] Rinaldi G, Hijazi A, Haghparast-Bidgoli H. Cost and cost-effectiveness of mHealth interventions for the prevention and control of type 2 diabetes mellitus: A systematic review. Diabetes Res ClinPract. 2020; 10.1016/j.diabres.2020.108084. 10.1016/j.diabres.2020.108084 32061819

[16] Paganini S, Teigelkötter W, Buntrock C, Baumeister H. Economic evaluations of internet- and mobile-based interventions for the treatment and prevention of depression: A systematic review. J Affect Disord. 2018; 10.1016/j.jad.2017.07.018. 10.1016/j.jad.2017.07.018 28922737

[17] Dubas-Jakóbczyk K, Kocot E, Kissimova-Skarbek K, Huter K, Rothgang H. Economic evaluation of health promotion and primary prevention actions for older people-a systematic review. Eur J Public Health. 2017; doi:10.1093/eurpub/ckx030. 10.1093/eurpub/ckx030 28371813

[18] Tate DF, Finkelstein EA, Khavjou O, Gustafson A, Tate DF, Finkelstein EA, et al. Cost effectiveness of internet interventions: review and recommendations. Ann Behav Med. 2009; 10.1007/s12160-009-9131-6. 10.1007/s12160-009-9131-6 PMC2772952 19834778

[19] Fischer F. [Digital interventions in prevention and health promotion: What kind of evidence do we have and what is needed?] Bundesgesundheitsblatt - Gesundheitsforschung - Gesundheitsschutz. 2020; 10.1007/s00103-020-03143-6. 10.1007/s00103-020-03143-6 32355991

[20] Page MJ, McKenzie JE, Bossuyt PM, Boutron I, Hoffmann TC, Mulrow CD, et al. The PRISMA 2020 statement: an updated guideline for reporting systematic reviews. BMJ. 2021; 10.1136/bmj.n71. 10.1136/bmj.n71 PMC8005924 33782057

[21] OECD. Purchasing power parities (PPP). 2021.https://www.oecd-ilibrary.org/content/data/1290ee5a-en. Accessed 10 Jan 2021.

[22] Husereau D, Drummond M, Petrou S, Carswell C, Moher D, Greenberg D, et al. Consolidated Health Economic Evaluation Reporting Standards (CHEERS)--explanation and elaboration. Value Health. 2013; 10.1016/j.jval.2013.02.002.

[23] Hubben GAA, Bos JM, Glynn DM, van der Ende A, van Alphen L, Postma MJ. Enhanced decision support for policy makers using a web interface to health-economic models - Illustrated with a cost-effectiveness analysis of nation-wide infant vaccination with the 7-valent pneumococcal conjugate vaccine in the Netherlands. Vaccine. 2007; 10.1016/j.vaccine.2007.01.088. 10.1016/j.vaccine.2007.01.088 17360082

[24] Luxton DD, Hansen RN, Stanfill K. Mobile app self-care versus in-office care for stress reduction: a cost minimization analysis. J Telemed Telecare. 2014; 10.1177/1357633x14555616. 10.1177/1357633X14555616 25316037

[25] Graves N, Barnett AG, Halton KA, Veerman JL, Winkler E, Owen N, et al. Cost-effectiveness of a telephone-delivered intervention for physical activity and diet. PLoS One. 2009; 10.1371/journal.pone.0007135. 10.1371/journal.pone.0007135 PMC2744997 19779611

[26] Pil L, Pauwels K, Muijzers E, Portzky G, Annemans L. Cost-effectiveness of a helpline for suicide prevention. J Telemed Telecare. 2013; 10.1177/1357633X13495487. 10.1177/1357633X13495487 24163237

[27] Smit F, Lokkerbol J, Riper H, Majo MC, Boon B, Blankers M (2011). Modeling the Cost-Effectiveness of Health Care Systems for Alcohol Use Disorders: How Implementation of eHealth Interventions Improves Cost-Effectiveness. J Med Internet Res.

[28] Smith KJ, Kuo S, Zgibor JC, McTigue KM, Hess R, Bhargava T, et al. Cost effectiveness of an internet-delivered lifestyle intervention in primary care patients with high cardiovascular risk. Prev Med. 2016;10.1016/j.ypmed.2016.02.036. 10.1016/j.ypmed.2016.02.036 26921656

[29] Patel B, Peiris DP, Patel A, Jan S, Harris MF, Usherwood T, et al. A computer-guided quality improvement tool for primary health care: cost-effectiveness analysis based on TORPEDO trial data. Med J Aust. 2020; 10.5694/mja2.50667. 10.5694/mja2.50667 32594567

[30] Burn E, Marshall AL, Miller YD, Barnett AG, Fjeldsoe BS, Graves N. The cost-effectiveness of the MobileMums intervention to increase physical activity among mothers with young children: a Markov model informed by a randomised controlled trial. BMJ Open. 2015; 10.1136/bmjopen-2014-007226. 10.1136/bmjopen-2014-007226 PMC4420940 25926145

[31] Cobiac LJ, Vos T, Barendregt JJ. Cost-Effectiveness of Interventions to Promote Physical Activity: A Modelling Study. PLoS Med. 2009; 10.1371/journal.pmed.1000110. 10.1371/journal.pmed.1000110 PMC2700960 19597537

[32] Peels DA, Hoogenveen RR, Feenstra TL, Golsteijn RH, Bolman C, Mudde AN, et al. Long-term health outcomes and cost-effectiveness of a computer-tailored physical activity intervention among people aged over fifty: modelling the results of a randomized controlled trial. BMC Public Health. 2014; 10.1186/1471-2458-14-1099. 10.1186/1471-2458-14-1099 PMC4221676 25342517

[33] Rondina R, Hong M, Sarma S, Mitchell M (2021). Is it worth it? Cost-effectiveness analysis of a commercial physical activity app. BMC Public Health.

[34] Mizdrak A, Telfer K, Direito A, Cobiac LJ, Blakely T, Cleghorn CL, et al. Health Gain, Cost Impacts, and Cost-Effectiveness of a Mass Media Campaign to Promote Smartphone Apps for Physical Activity: Modeling Study. J Med Internet Res. 2020; 10.2196/18014. 10.2196/18014 PMC7317635 32525493

[35] Goryakin Y, Aldea A, Lerouge A, Romano Spica V, Nante N, Vuik S, et al. Promoting sport and physical activity in Italy: a cost-effectiveness analysis of seven innovative public health policies. Ann Ig. 2019; 10.7416/ai.2019.2321. 10.7416/ai.2019.2321 31616905

[36] Miners A, Harris J, Felix L, Murray E, Michie S, Edwards P. An economic evaluation of adaptive e-learning devices to promote weight loss via dietary change for people with obesity. BMC Health Serv Res. 2012; 10.1186/1472-6963-12-190. 10.1186/1472-6963-12-190 PMC3438094 22769737

[37] Cleghorn C, Wilson N, Nair N, Kvizhinadze G, Nghiem N, McLeod M, et al. Health Benefits and Cost-Effectiveness From Promoting Smartphone Apps for Weight Loss: Multistate Life Table Modeling. JMIR Mhealth and Uhealth. 2019; 10.2196/11118. 10.2196/11118 PMC6350086 30664471

[38] Jones AC, Grout L, Wilson N, Nghiem N, Cleghorn C. The Cost-effectiveness of a Mass Media Campaign to Promote Smartphone Apps for Weight Loss: Updated Modeling Study. JMIR Form Res. 2022; 10.2196/29291. 10.2196/29291 PMC9066337 35438643

[39] Guerriero C, Cairns J, Roberts I, Rodgers A, Whittaker R, Free C. The cost-effectiveness of smoking cessation support delivered by mobile phone text messaging: Txt2stop. Eur J Health Econ. 2013; 10.1007/s10198-012-0424-5. 10.1007/s10198-012-0424-5 PMC3751449 22961230

[40] Cobos-Campos R, Mar J, Apinaniz A, de Lafuente AS, Parraza N, Aizpuru F, et al. Cost-effectiveness analysis of text messaging to support health advice for smoking cessation. Cost Eff Resour Alloc. 2021; 10.1186/s12962-021-00262-y. 10.1186/s12962-021-00262-y PMC7885425 33588885

[41] Cheung KL, Wijnen BFM, Hiligsmann M, Coyle K, Coyle D, Pokhrel S, et al. Is it cost-effective to provide internet-based interventions to complement the current provision of smoking cessation services in the Netherlands? An analysis based on the EQUIPTMOD. Addiction. 2018; 10.1111/add.14069. 10.1111/add.14069 PMC6032907 29243351

[42] Kruger J, Brennan A, Strong M, Thomas C, Norman P, Epton T. The cost-effectiveness of a theory-based online health behaviour intervention for new university students: an economic evaluation. BMC Public Health. 2014; 10.1186/1471-2458-14-1011. 10.1186/1471-2458-14-1011 PMC4195974 25262372

[43] Song M, Kanaoka H. Effectiveness of mobile application for menstrual management of working women in Japan: randomized controlled trial and medical economic evaluation. J Med Econ. 2018; 10.1080/13696998.2018.1515082. 10.1080/13696998.2018.1515082 30130990

[44] Culyer A, McCabe C, Briggs A, Claxton K, Buxton M, Akehurst R, et al. Searching for a threshold, not setting one: the role of the National Institute for Health and Clinical Excellence. J Health Serv Res Policy. 2007; 10.1258/135581907779497567. 10.1258/135581907779497567 17244400

[45] Tate DF, Finkelstein EA, Khavjou O, Gustafson A. Cost Effectiveness of Internet Interventions: Review and Recommendations. Ann Behav Med. 2009; 10.1007/s12160-009-9131-6. 10.1007/s12160-009-9131-6 PMC2772952 19834778

[46] de la Torre-Diez I, Lopez-Coronado M, Vaca C, Aguado JS, de Castro C. Cost-Utility and Cost-Effectiveness Studies of Telemedicine, Electronic, and Mobile Health Systems in the Literature: A Systematic Review. Telemedicine and E-Health. 2015; 10.1089/tmj.2014.0053. 10.1089/tmj.2014.0053 PMC4312789 25474190

[47] Ghani Z, Jarl J, Berglund JS, Andersson M, Anderberg P. The Cost-Effectiveness of Mobile Health (mHealth) Interventions for Older Adults: Systematic Review. Int J Environ Res Public Health. 2020; 10.3390/ijerph17155290. 10.3390/ijerph17155290 PMC7432315 32708016

[48] Sanyal C, Stolee P, Juzwishin D, Husereau D. Economic evaluations of eHealth technologies: A systematic review. PLoS One. 2018; 10.1371/journal.pone.0198112. 10.1371/journal.pone.0198112 PMC5999277 29897921

[49] Iribarren SJ, Cato K, Falzon L, Stone PW. What is the economic evidence for mHealth? A systematic review of economic evaluations of mHealth solutions. PLoS One. 2017; 10.1371/journal.pone.0170581. 10.1371/journal.pone.0170581 PMC5289471 28152012

[50] Law L, Kelly JT, Savill H, Wallen MP, Hickman IJ, Erku D, et al. Cost-effectiveness of telehealth-delivered diet and exercise interventions: A systematic review. J Telemed Telecare. 2022; 10.1177/1357633x211070721. 10.1177/1357633X211070721 35108135

[51] Kravitz R, Duan N DMCN-o-GP. Design and Implementation of N-of-1 Trials: A User’s Guide. 2014. https://effectivehealthcare.ahrq.gov/sites/default/files/pdf/n-1-trials_research-2014-5.pdf. Accessed 19 Sept 2022.

[52] Haddix AC, Teutsch SM, Corso PS. Prevention effectiveness: a guide to decision analysis and economic evaluation: Oxford University Press; 2002.

[53] Agrawal S, Wojtanowski AC, Tringali L, Foster GD, Finkelstein EA. Financial implications of New York City's weight management initiative. PLoS One. 2021; 10.1371/journal.pone.0246621. 10.1371/journal.pone.0246621 PMC7877753 33571249

[54] Zurovac D, Larson BA, Sudoi RK, Snow RW. Costs and Cost-Effectiveness of a Mobile Phone Text-Message Reminder Programmes to Improve Health Workers' Adherence to Malaria Guidelines in Kenya. PLoS One. 2012; 10.1371/journal.pone.0052045. 10.1371/journal.pone.0052045 PMC3525566 23272206

[55] Shields GE, Elvidge J. Challenges in synthesising cost-effectiveness estimates. Systematic Reviews. 2020; 10.1186/s13643-020-01536-x. 10.1186/s13643-020-01536-x PMC7727163 33298168

[56] Philips Z, Bojke L, Sculpher M, Claxton K, Golder S (2006). Good practice guidelines for decision-analytic modelling in health technology assessment. Pharmacoeconomics.

[57] Lange O, Plath J, Dziggel TF, Karpa DF, Keil M, Becker T (2022). A Transparency Checklist for Carbon Footprint Calculations Applied within a Systematic Review of Virtual Care Interventions. Int J Environ Res Public Health.

