# Supplementary material for: Health economic evaluation of preventive digital public health interventions using decision-analytic modelling: a systematized review
Source: BMC Health Serv Res. 2023 Mar 17;23:268. doi: 10.1186/s12913-023-09280-3 (PMC10024449; doi:10.1186/s12913-023-09280-3)
Supplement: Supplementary file 1 — Additional file 1. [file 12913_2023_9280_MOESM1_ESM.pdf]

Additional file 1: Seach Strategy

**Database**	**Date**	**Search**	**Hits**
PubMed	04.12.2020	("app"[Title/Abstract] OR "apps"[Title/Abstract] OR "smartphone"[Title/Abstract] OR "mobile phone"[Title/Abstract] OR "Smartwatch"[Title/Abstract] OR "cellphone"[Title/Abstract] OR "internet"[Title/Abstract] OR "online"[Title/Abstract] OR "website"[Title/Abstract] OR "web-based" OR "fitness tracker"[Title/Abstract] OR "fitnesstracker"[Title/Abstract] OR "acceleromet*"[Title/Abstract] OR "activity monitor"[Title/Abstract] OR "digital health"[Title/Abstract] OR "digital public health"[Title/Abstract] OR "digital intervention*"[Title/Abstract] OR uhealth[Title/Abstract] OR u-health[Title/Abstract] OR m-health[Title/Abstract] OR e-health[Title/Abstract] OR ehealth[Title/Abstract] OR mhealth[Title/Abstract] OR "Mobile health"[Title/Abstract] OR telemedicine OR telehealth[Title/Abstract] OR Telecare[Title/Abstract]) AND ("cost-effectiveness"[title] OR "cost-benefit"[title] OR "cost-utility"[title] OR "cost-minimization"[title] OR "cost-minimisation"[title] OR "cost-consequences"[title] OR "cost-consequence"[title] OR "economic evaluation"[title])	970
PubMed - Search details	-	("app"[Title/Abstract] OR "apps"[Title/Abstract] OR "smartphone"[Title/Abstract] OR "mobile phone"[Title/Abstract] OR "Smartwatch"[Title/Abstract] OR "cellphone"[Title/Abstract] OR "internet"[Title/Abstract] OR "online"[Title/Abstract] OR "website"[Title/Abstract] OR "web-based"[All Fields] OR "fitness tracker"[Title/Abstract] OR "fitnesstracker"[Title/Abstract] OR "acceleromet*"[Title/Abstract] OR "activity monitor"[Title/Abstract] OR "digital health"[Title/Abstract] OR "digital public health"[Title/Abstract] OR "digital intervention*"[Title/Abstract] OR "uhealth"[Title/Abstract] OR "u-health"[Title/Abstract] OR "m-health"[Title/Abstract] OR "e-health"[Title/Abstract] OR "ehealth"[Title/Abstract] OR "mhealth"[Title/Abstract] OR "Mobile health"[Title/Abstract] OR ("telemedicine"[MeSH Terms] OR "telemedicine"[All Fields] OR "telemedicine s"[All Fields]) OR "telehealth"[Title/Abstract] OR "Telecare"[Title/Abstract]) AND ("cost-effectiveness"[Title] OR "cost-benefit"[Title] OR "cost-utility"[Title] OR "cost-minimization"[Title] OR "cost-minimisation"[Title] OR "cost-consequences"[Title] OR "cost-consequence"[Title] OR "economic evaluation"[Title])	-
CINAHL	04.12.2020	(TI (("app" OR "apps" OR "smartphone" OR "mobile phone" OR "Smartwatch" OR "cellphone" OR "internet" OR "online" OR "website" OR "web-based" OR "fitness tracker" OR "fitnesstracker" OR "acceleromet*" OR "activity monitor" OR (digital health) OR "digital public health" OR "digital intervention*" OR uhealth OR u-health OR m-health OR e-health OR ehealth OR mhealth OR (Mobile health) OR telemedicine OR telehealth OR Telecare)) OR AB (("app" OR "apps" OR "smartphone" OR "mobile phone" OR "Smartwatch" OR "cellphone" OR "internet" OR "online" OR "website" OR "web-based" OR "fitness tracker" OR "fitnesstracker" OR "acceleromet*" OR "activity monitor" OR (digital health) OR "digital public health" OR "digital intervention*" OR uhealth OR u-health OR m-health OR e-health OR ehealth OR mhealth OR (Mobile health) OR telemedicine OR telehealth OR Telecare))) AND TI (("cost-effectiveness“ OR „cost-benefit“ OR „cost-utility“ OR „cost-minimization“ OR „cost-minimisation“ OR „cost-consequences“ OR "cost-consequence" OR „economic evaluation“))	314
Web of Science	04.12.2020	(TI=("app" OR "apps" OR "smartphone" OR "mobile phone" OR "Smartwatch" OR "cellphone" OR "internet" OR "online" OR "website" OR "web-based" OR "fitness tracker" OR "fitnesstracker" OR "acceleromet*" OR "activity monitor" OR (digital health) OR "digital public health" OR "digital intervention*" OR uhealth OR u-health OR m-health OR e-health OR ehealth OR mhealth OR (Mobile health) OR telemedicine OR telehealth OR Telecare) OR AB=("app" OR "apps" OR "smartphone" OR "mobile phone" OR "Smartwatch" OR "cellphone" OR "internet" OR "online" OR "website" OR "web-based" OR "fitness tracker" OR "fitnesstracker" OR "acceleromet*" OR "activity monitor" OR (digital health) OR "digital public health" OR "digital intervention*" OR uhealth OR u-health OR m-health OR e-health OR ehealth OR mhealth OR (Mobile health) OR telemedicine OR telehealth OR Telecare)) AND TI=("cost-effectiveness“ OR „cost-benefit“ OR „cost-utility“ OR „cost-minimization“ OR „cost-minimisation“ OR „cost-consequences“ OR "cost-consequence" OR „economic evaluation“)	654

Search Update

PubMed	01.06.2022 (Search Update)	(("app"[Title/Abstract] OR "apps"[Title/Abstract] OR "smartphone"[Title/Abstract] OR "mobile phone"[Title/Abstract] OR "Smartwatch"[Title/Abstract] OR "cellphone"[Title/Abstract] OR "internet"[Title/Abstract] OR "online"[Title/Abstract] OR "website"[Title/Abstract] OR "web-based" OR "fitness tracker"[Title/Abstract] OR "fitnesstracker"[Title/Abstract] OR "acceleromet*"[Title/Abstract] OR "activity monitor"[Title/Abstract] OR "digital health"[Title/Abstract] OR "digital public health"[Title/Abstract] OR "digital intervention*"[Title/Abstract] OR uhealth[Title/Abstract] OR u-health[Title/Abstract] OR m-health[Title/Abstract] OR e-health[Title/Abstract] OR ehealth[Title/Abstract] OR mhealth[Title/Abstract] OR "Mobile health"[Title/Abstract] OR telemedicine OR telehealth[Title/Abstract] OR Telecare[Title/Abstract]) AND ("cost-effectiveness"[title] OR "cost-benefit"[title] OR "cost-utility"[title] OR "cost-minimization"[title] OR "cost-minimisation"[title] OR "cost-consequences"[title] OR "cost-consequence"[title] OR "economic evaluation"[title])) AND **(("2020/12/05"[Date - Entry] : "2022/06/01"[Date - Entry]))**	210
CINAHL	01.06.2022 (Search Update)	(TI (("app" OR "apps" OR "smartphone" OR "mobile phone" OR "Smartwatch" OR "cellphone" OR "internet" OR "online" OR "website" OR "web-based" OR "fitness tracker" OR "fitnesstracker" OR "acceleromet*" OR "activity monitor" OR (digital health) OR "digital public health" OR "digital intervention*" OR uhealth OR u-health OR m-health OR e-health OR ehealth OR mhealth OR (Mobile health) OR telemedicine OR telehealth OR Telecare)) OR AB (("app" OR "apps" OR "smartphone" OR "mobile phone" OR "Smartwatch" OR "cellphone" OR "internet" OR "online" OR "website" OR "web-based" OR "fitness tracker" OR "fitnesstracker" OR "acceleromet*" OR "activity monitor" OR (digital health) OR "digital public health" OR "digital intervention*" OR uhealth OR u-health OR m-health OR e-health OR ehealth OR mhealth OR (Mobile health) OR telemedicine OR telehealth OR Telecare))) AND TI (("cost-effectiveness“ OR „cost-benefit“ OR „cost-utility“ OR „cost-minimization“ OR „cost-minimisation“ OR „cost-consequences“ OR "cost-consequence" OR „economic evaluation“)) AND **(EM 20201205-20220601)**	37
Web of Science	01.06.2022 (Search Update)	(TI=("app" OR "apps" OR "smartphone" OR "mobile phone" OR "Smartwatch" OR "cellphone" OR "internet" OR "online" OR "website" OR "web-based" OR "fitness tracker" OR "fitnesstracker" OR "acceleromet*" OR "activity monitor" OR (digital health) OR "digital public health" OR "digital intervention*" OR uhealth OR u-health OR m-health OR e-health OR ehealth OR mhealth OR (Mobile health) OR telemedicine OR telehealth OR Telecare) OR AB=("app" OR "apps" OR "smartphone" OR "mobile phone" OR "Smartwatch" OR "cellphone" OR "internet" OR "online" OR "website" OR "web-based" OR "fitness tracker" OR "fitnesstracker" OR "acceleromet*" OR "activity monitor" OR (digital health) OR "digital public health" OR "digital intervention*" OR uhealth OR u-health OR m-health OR e-health OR ehealth OR mhealth OR (Mobile health) OR telemedicine OR telehealth OR Telecare)) AND TI=("cost-effectiveness“ OR „cost-benefit“ OR „cost-utility“ OR „cost-minimization“ OR „cost-minimisation“ OR „cost-consequences“ OR "cost-consequence" OR „economic evaluation“)<br>**Timespan: 2020-12-05 to 2022-01-06 (Index Date)**	87
