# Supplementary material for: Health economic evaluation of preventive digital public health interventions using decision-analytic modelling: a systematized review
Source: BMC Health Serv Res. 2023 Mar 17;23:268. doi: 10.1186/s12913-023-09280-3 (PMC10024449; doi:10.1186/s12913-023-09280-3)
Supplement: Supplementary file 2 — Additional file 2. [file 12913_2023_9280_MOESM2_ESM.pdf]

Additional file 2: Study characteristics

	Type of economic evaluation	Model	Primary health outcome	Time horizon	Discount rate outcome	Discount rate cost	Measurement of effectiveness	Aim	Focused digital aspect	Complementary digital tools	Face-to-face required	Target population	Location	Study perspective	Funding
Burn et al. [30]	CUA	Markov model	QALYs	Short term (2 years)	5%	5%	Single-study based	Physical activity	Text-messages	Web-based tools	Yes	Woman with < 1 year old children	Australia	Health service perspective	Public funding
Cheung et al. [41]	CUA	Markov model	QALYs	Lifetime, 10-year, 5-year, 2-year	1.5%	4.0%	Synthesis-based	Smoking cessation	Web-based (Personalized feedback)	Not identified	Intervention is evaluated in addition to standard care	Smoker	Netherlands	Health care perspective	Public funding
Cleghorn et al. [37]	CUA	Multistate life table	QALYs	Lifetime	3%	3%	Synthesis-based	Weight loss	Promotion for app	Not identified	Not identified	Overweight adults	New Zealand	Health system perspective	Public funding
Cobiac et al. [31]	CUA	Multistate life table	DALYs	Lifetime	3%	3%	Synthesis-based	Physical activity	Mixed internet-based	Not identified	Not identified	Adult general population	Australia	Health sector perspective	Public funding
Goryakin et al. [35]	CUA	Micro-simulation model	DALYs	Medium term (2019-2050)	3%	3%	Synthesis-based	Weight loss	App-based intervention	Not identified	Not identified	Adult general population	Italy	Health system perspective	Public funding
Guerriero et al. [Only Mobile App intervention] [39]	CEA+CUA	Markov model	QALYs	Lifetime	3.5%	3.5%	Synthesis-based	Smoking cessation	Text-messages	Not identified	Not identified	Smoker	UK	Health service perspective	Public funding
Jones et al. [38]	CUA	Multistate life table	QALYs	Lifetime	3%	3%	Single-study based	Weightloss	Smartphone-App (Promotion)	Not identified	Not identified	New Zealand adult population	New Zealand	Health system perspective	Public funding
Kruger et al. [42]	CEA+CUA	Markov model	QALYs	Lifetime	1.5%	Not identified	Synthesis-based	Behaviour change (mixed)	Web-based	Not identified	Not identified	Young people starting university	UK	UK Department of Health	Public funding
Miners et al. [36]	CUA	Discrete Event Simulation	QALYs	Lifetime	3.5%	3.5%	Synthesis-based	Weight loss	E-Learning Device	Not identified	Not identified	Overweight adults	UK	Health service perspective	Public funding
Mizdrak et al. [34]	CUA	Multistate life table	QALYs	Lifetime	3%	3%	Synthesis-based	Physical activity	Promotion for app	Not identified	Not identified	Adult general population	New Zealand	Health system perspective	Public funding
Peels et al. [32]	CEA+CUA	Markov model	QALYs	Lifetime, 10-year, 5-year	1.5%	4%	Single-study based	Physical activity	Web-based	Web-based tools	Not identified	> 50 years old person	Netherlands	Health care perspective	Public funding
Cobos-Campos [40]	CUA	Markov model	QALYs	Life-time	3%	3%	Study-based	Smoking cessation	Text messages	Not identified	Yes, Health advice	Smoker	Spain	Health system + productivity losses	Public funding
Rondina et al. [33]	CUA	Markov model	QALYs	5 year	1.5%	1.5%	Single-study based	Physical activity	Smartphone App	Not identified	Not identified	Youth & Adults	Canada	Public healthcare payer	Mixed
Song et al. [43]	CUA	Discrete Event Simulation	QALYs	Medium term (Until the end of age 45)	2%	2%	Single-study based	Menstrual management	App-based intervention	Not identified	Not identified	Woman aged 20-45 years	Japan	Health insurance + productivity losses	Industrial funding
